# Application of Temperature-Dependent Fluorescent Dyes to the Measurement of Millimeter Wave Absorption in Water Applied to Biomedical Experiments

**DOI:** 10.1155/2014/243564

**Published:** 2014-11-11

**Authors:** Nataliia Kuzkova, Oleksandr Popenko, Andrey Yakunov

**Affiliations:** Physics Department, Taras Shevchenko National University of Kyiv, Building No. 1, 4 Glushkova Avenue, Kiev 03127, Ukraine

## Abstract

Temperature sensitivity of the fluorescence intensity of the organic dyes solutions was used for noncontact measurement of the electromagnetic millimeter wave absorption in water. By using two different dyes with opposite temperature effects, local temperature increase in the capillary that is placed inside a rectangular waveguide in which millimeter waves propagate was defined. The application of this noncontact temperature sensing is a simple and novel method to detect temperature change in small biological objects.

## 1. Introduction

Biological effects of electromagnetic millimeter waves were observed in many experiments on various biological objects, starting from the bacteria to the whole human body as well as model systems in general [1]. It was repeatedly noted that the nature of the microwaves on biological objects is different from the conventional thermal effect of electromagnetic waves of the other bands, and the physical nature of this phenomenon is still unclear. In order to implement adequate physical model an actual definition of “non-thermal effect” requires precise temperature measurement of irradiated objects.

It is considered that the use of low power (with output power up to 20 mW) millimeter waves generators does not result into significant heating of the irradiated matter. Calculations [2] provide an increase of temperature to 1°C for different patterns of exposure, which should not cause essential biological effects. However, direct measurement of the temperature change in the area of irradiation is a challenging technical problem. High-sensitivity temperature sensors (thermocouples, thermistors, etc.), making perturbations in the investigated samples, affect the local temperature and heat transfer characteristics in a given place. Although less accurate, optical methods based on visible or ultraviolet absorption, infrared, Raman, and fluorescence spectroscopy are more convenient for registration of temperature changes in small volumes [3]. In contrast, fluorescence-based thermometry which has satisfactory metrological parameters (sensitivity, performance, spatial resolution) at the same time is simple and reliable.

The water in the millimeter wave range is characterized by a considerable absorption of electromagnetic waves. In particular, at a frequency of 50 GHz, corresponding to wavelengths in vacuum of 6 mm, the absorption coefficient is equal to 5.1 mm^−1^ [2]. Accordingly, the spatial temperature distribution in the irradiated samples containing water is very inhomogeneous. Electromagnetic millimeter waves heat the substances containing water in a thin surface layer with high temperature gradient, the numerical value of which can be estimated only by indirect methods. Therefore, existing methods for irradiation of biological objects are imperfect and metrological control tools are not reliable.

To determine temperature changes in water caused by the millimeter wave absorption we used an optical noncontact method, which is based on the existence of the fluorescence intensity dependence of temperature of organic dyes. The local temperature rise in the capillary was measured, placed inside a rectangular waveguide, in which millimeter waves propagate. We have chosen two dyes with opposite temperature effects: Rhodamine 6G (R6G) and Rhodamine C (RC).

## 2. Methodology and Experimental Measurements 

Calibration of the fluorescence intensity of organic dyes aqueous solutions on temperature was carried out by means of diffraction spectrometer. Semiconductor laser emission with 406 nm wavelength and 60 mW output power was used as an excitation source. The samples were prepared from standard distilled water for medical purpose. The concentration of dyes (~0.4 g/liter) was chosen from the condition of maximum temperature sensitivity and temperature coefficients of approximate equality [3].

A straight-through scheme for illumination and viewing was used [4]. The glass cuvette (3 mm pass length) with the dye solution was placed inside a large glass rectangular cell with water, which served as a water bath. Water temperature was maintained to within 0.2°.

**Figure 1:**
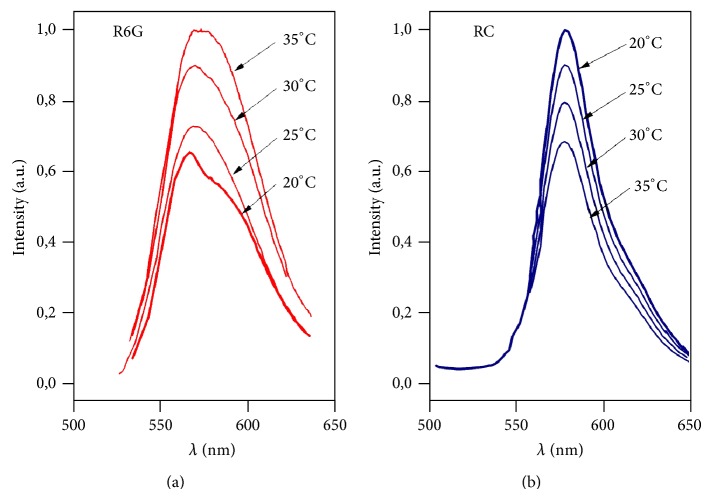
Fluorescence spectrum of Rhodamine 6G (R6G) and Rhodamine C (RC) at 5°C intervals from 20 to 35°C.

**Figure 2:**
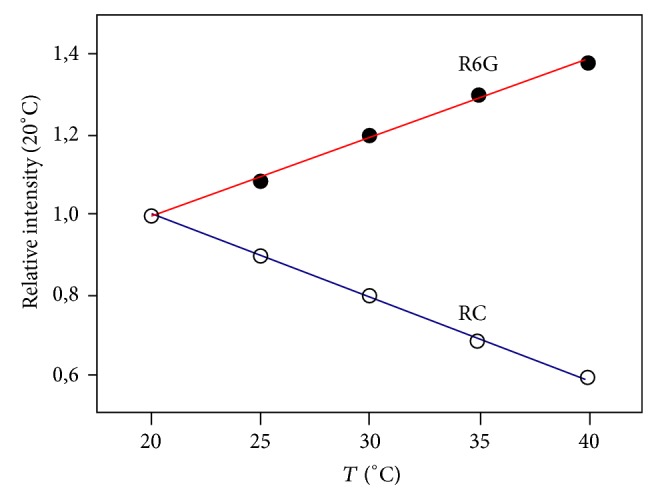
Temperature dependence of the relative fluorescence intensity of R6G and RC.

The fluorescence spectra were measured at temperatures from 20°C to 40°C, over each 5°C (Figure 1). The intensity of fluorescence was normalized by the initial value, which corresponded to the temperature of 20°C (Figure 2).

It was established that, in most cases, the temperature rise is making destructive contribution to the fluorescence yield [3]. With the growth of temperature, the frequency and energy of molecules collisions in solution, as well as the amplitude of internal molecular vibrations, increase, leading to an increase in nonradiative relaxation of the excited levels, and thus fluorescence quenching. Along with the temperature quenching of the dye solution there are possible mechanisms that increase the overall yield of fluorescence with increasing temperature [5]. In particular, some organic molecules in aqueous solution tend to form associated complexes: dimers, trimers, and so forth, where fluorescence quantum yield is much lower than that in the individual molecules. At sufficiently high concentrations, the fluorescence spectrum is formed as a superposition of the spectra of individual molecules and their associates. Some associates divided into separate molecules with increasing temperature that is accompanied by a relative increase in fluorescence intensity.

Figure 2 shows that the fluorescence temperature dependence of the RC solution corresponds to the one option, while R6G to another, and in the range 20 ÷ 40°C their intensities approximated by a linear function: (*T*)/*I* (20°C) = *a* + *bT*, where *b* is a temperature sensitivity coefficient. For given concentrations of solutions this parameter is equal to *b*_R6G_ = 0.019°C^−1^ and *b*_RC_ = −0.021°C^−1^.

**Figure 3:**
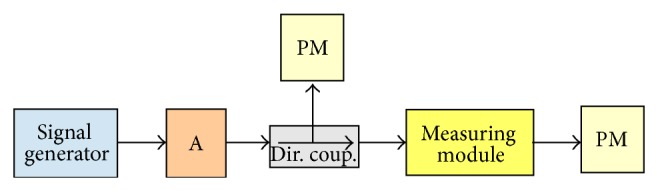
Schematic of experimental set-up. Signal generator: A: attenuator; Dir. coup.: directional coupler; measuring module; PM: power meter.

**Figure 4:**
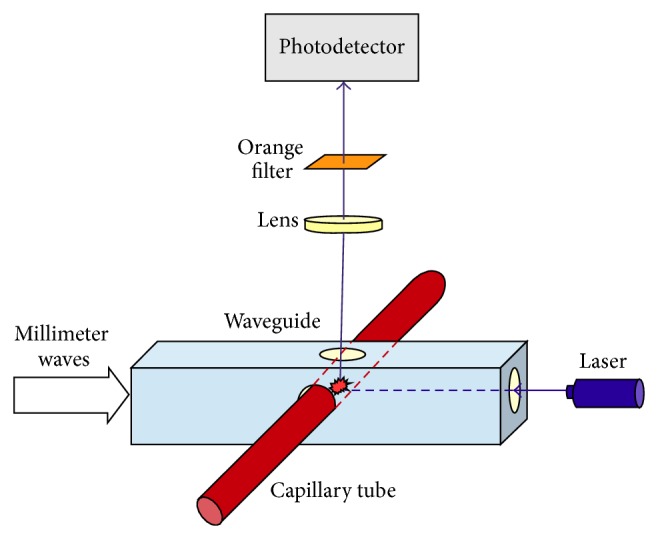
The measuring module is composed of laser, focusing lenses, glass capillary with a dye solution, waveguide, optical filter, and photodetector.

Schematic of the experimental set-up is shown in Figure 3. Millimeter wave radiation from high frequency signal generator through polarizing attenuator was applied to the measuring module (Figure 4). Attenuator is included to prevent reflected power from damaging the amplifier. The measuring module consists of the segment of rectangular waveguide with a cross section 7.2 × 3.4 mm, inside which, through the middle, was inserted a glass capillary with an inner diameter of 0.5 mm. Focused laser beam excited fluorescence in filled capillary of dye solution.

For capillary with water in a rectangular waveguide area of maximum absorption of the electromagnetic millimeter wave corresponds to antinodes of electrical components and is concentrated in the central part of Δ*x* ~ 1 mm [6]. Fluorescence signal from the central section was removed through a round hole made in the middle of the narrow waveguide wall opposite the capillary. The light stream focused by lens and passed through an orange filter to a photodetector.

Some radiation that was applied to the measurement module through waveguide directional coupler (Figure 4) fell on power meter and has been registered as a signal proportional to the input power (*α* · *P*_in_), where *α* = 0.1 is coefficient coupler. Similarly, the radiation that passes through the module was recorded as a signal proportional to the output power (*α* · *P*_out_). The difference Δ*P* = *P*_in_ − *P*_out_ was equal to the power, absorbed in the sample. Previously in the range of 40–50 GHz frequency dependence of the capillary absorption of water was measured. At a frequency of 47.5 GHz a pronounced resonance maximum was observed, typical for rectangular waveguide with dielectric cylinder of small diameter [6].

Further experiments were carried out at the frequency of resonance. Power was absorbed into the capillary with a solution governed by polarization attenuator with attenuation coefficients 0, 1, 2, 3, 4 dB, which corresponded to an absolute value of 20 mW, 15.8 mW, 12.5 mW, 10 mW, and 7.9 mW, respectively.

Duration of registration signal *T* ≈ 40 s was chosen from the condition significantly exceeded time of establishing thermal equilibrium: *T* ≫ *τ*. Constant of relaxation was assessed by the expression *τ* ~ (Δ*x*)^2^/*D*, where *D* is the coefficient of thermal conductivity of water was of the order of a few seconds for a given irradiation scheme.

## 3. Results and Analysis 

**Figure 5:**
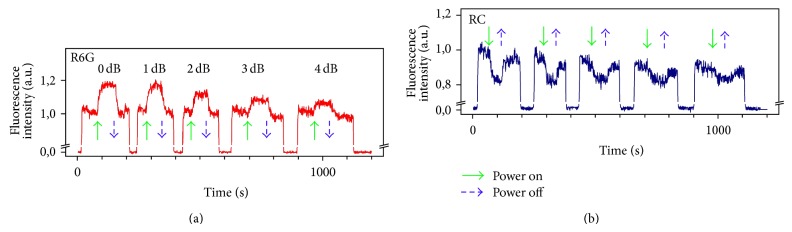
Dynamics of the change in fluorescence intensity when millimeter wave radiation is turned on and turned off. The voltage is switched off and the fluorescence returns to the base line before a different voltage is applied to the microheater. The two curves illustrate the repeatability of the fluorescence to achieve the same level for a given temperature.

Figure 5 shows the dynamics of R6G and PC fluorescence dyes for switched on and off microwave radiation with different capacities. The absorption of microwaves leads to fluorescence increasing for R6G solution and fluorescence quenching for RC. The gradual decrease in fluorescence intensity is caused by photodestruction of organic dye molecules. By switching off temporarily laser emission, the initial level of fluorescence was almost restored.

**Figure 6:**
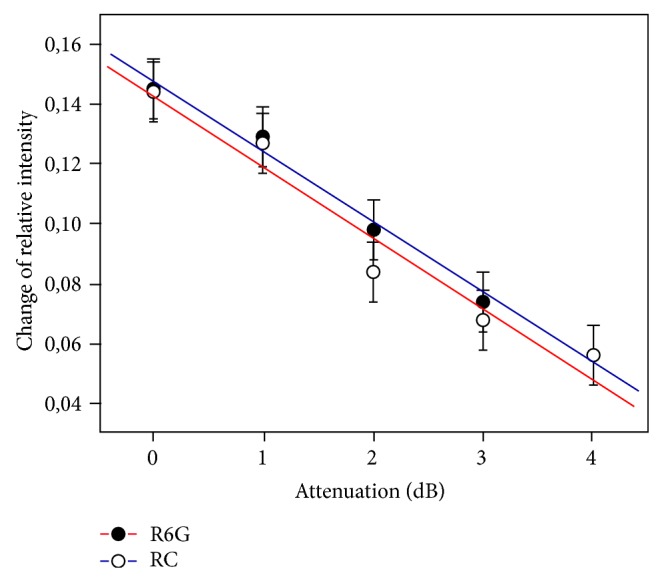
Change of relative fluorescence intensity of the relaxation factor of attenuator.

The dependence of the relative change on fluorescence intensity |*I* − *I*_20_|/*I* of the absorbed power in the capillary is obtained from the experimental data (Figure 6). In the first approximation it can be approximated by linear functions:

(1) $$\begin{array}{c} y=0.15-0.023x\;\text{for}\;\text{R}6\text{G};\;\\ y=0.14-0.024x\;\text{for}\;\text{RC}. \end{array}$$

Thus taking into account the calibration dependence (Figure 2), it corresponds to a local temperature increase in the capillary absorbed at maximum power 20 mW: Δ*T*_1_ = (7.7 ± 0.4)°C for R6G and Δ*T*_2_ = (7.4 ± 0.4)°C for RC.

It could be seen that even a low-power microwave source, under certain conditions, causes significant heating of the sample, which can lead to significant biological effects. It should be noted that the special conditions of the experiment (placing the sample inside the waveguide, radiation at the resonant frequency) determine the maximum possible temperature response.

Although in the vast majority of biophysical experiments irradiation conditions of millimeter wave are not so tough, however are carried out in the near zone of the horn or waveguide. In the vicinity of the irradiated object there are standing waves that form parasitic resonances. The appearance of the local area temperature increase of a few degrees can cause thermal biological effects at low levels of total radiation power.

It is assumed [7, 8] that millimeter wave irradiation of dyes solutions has possible additional nonthermal effect of millimeter waves on the spectral properties of solutions which is due to the change in the structure of water. During irradiation, pseudo polymer hydrogen bonding network undergoes deformation, rearrangement, and perhaps destruction. Restructuring of water under millimeter wave field causes structural changes in the molecular associates and promotes their decay.

In our study, we obtained the same value of the effective temperature for solutions with different scenarios of temperature behavior. This indicates the absence of nonthermal effect of millimeter waves on water, at least in the conditions of sample irradiation inside the resonator at the resonant frequency.

Moreover, comparison of dependency in Figure 4 leads to the same conclusion. The equality of slope coefficients approximating lines indicates the fact that probable nonthermal effect of millimeter waves on water under the given conditions can be neglected.

## 4. Conclusions

Optical noncontact method, which is based on the existence of the fluorescence intensity temperature dependence of the organic dyes, was used to determine changes in water temperature in the millimeter wave absorption.

Using two dyes with opposite effects, temperature heating an aqueous solution in the capillary was measured, placed inside a rectangular waveguide.

Using even low-power sources of millimeter waves, the local increase in temperature of the sample can be several degrees. At the same time, the results indicate the absence of nonthermal effect of millimeter waves on the water.

The application of noncontact temperature sensing based on the temperature sensitivity of florescent dyes is a simple and novel method to detect temperature change in small biological objects.
